# Tuberculosis

**Published:** 1909-12

**Authors:** 


**Tuberculosis.** A Treatise by American Authors. Edited by **Arnold C. Klebs.** Illustrated. Octavo, pp. 939. New York and London: D. Appleton & Co. (Cloth, $6.00.)

This treatise begins with an historical introduction by Professor Osler, and presents a systematic discussion of the whole subject of tuberculosis in the light of our present knowledge. The editor states in his preface that in view of the enormous amount of literature on the subject of tuberculosis that has appeared during the past year, a consideration of the entire subject by one author is impossible and inexpedient. For that reason every chapter deals with a distinct phase of the subject. Eighteen contributors give their special views, lending interest to the subject.

Taken as a whole, the book shows an enormous amount of research. Contains a vast amount of information, and is one of the most authoritative and valuable expositions on the subjects in any language. In his statistical figures and tables, on pages 114, 115 and 116, Klebs gives a wonderfully clear description of prevailing conditions and ends with the remark "Tuberculosis constitutes the gravest social problem which is now engaging the attention of the whole civilised world in a common effort against it. When we take into consideration the fact that in tuberculosis we have a deadlier enemy among us, than wars, earthquakes and floods, that the victims of this scourge during the past three years in the United States are greater in number than the entire population of Buffalo and Rochester combined, and if the line of consumptive dead for one year were stretched out one after the other, without intervening space, it would reach from New York to Boston. We can then well agree with the editor, that it is a world problem, and its settlement is in the distant future.

